# Supplementary material for: BLM helicase suppresses recombination at G-quadruplex motifs in transcribed genes
Source: Nat Commun. 2018 Jan 18;9:271. doi: 10.1038/s41467-017-02760-1 (PMC5773480; doi:10.1038/s41467-017-02760-1)
Supplement: Supplementary file 1 — Supplementary Information [file 41467_2017_2760_MOESM1_ESM.pdf]

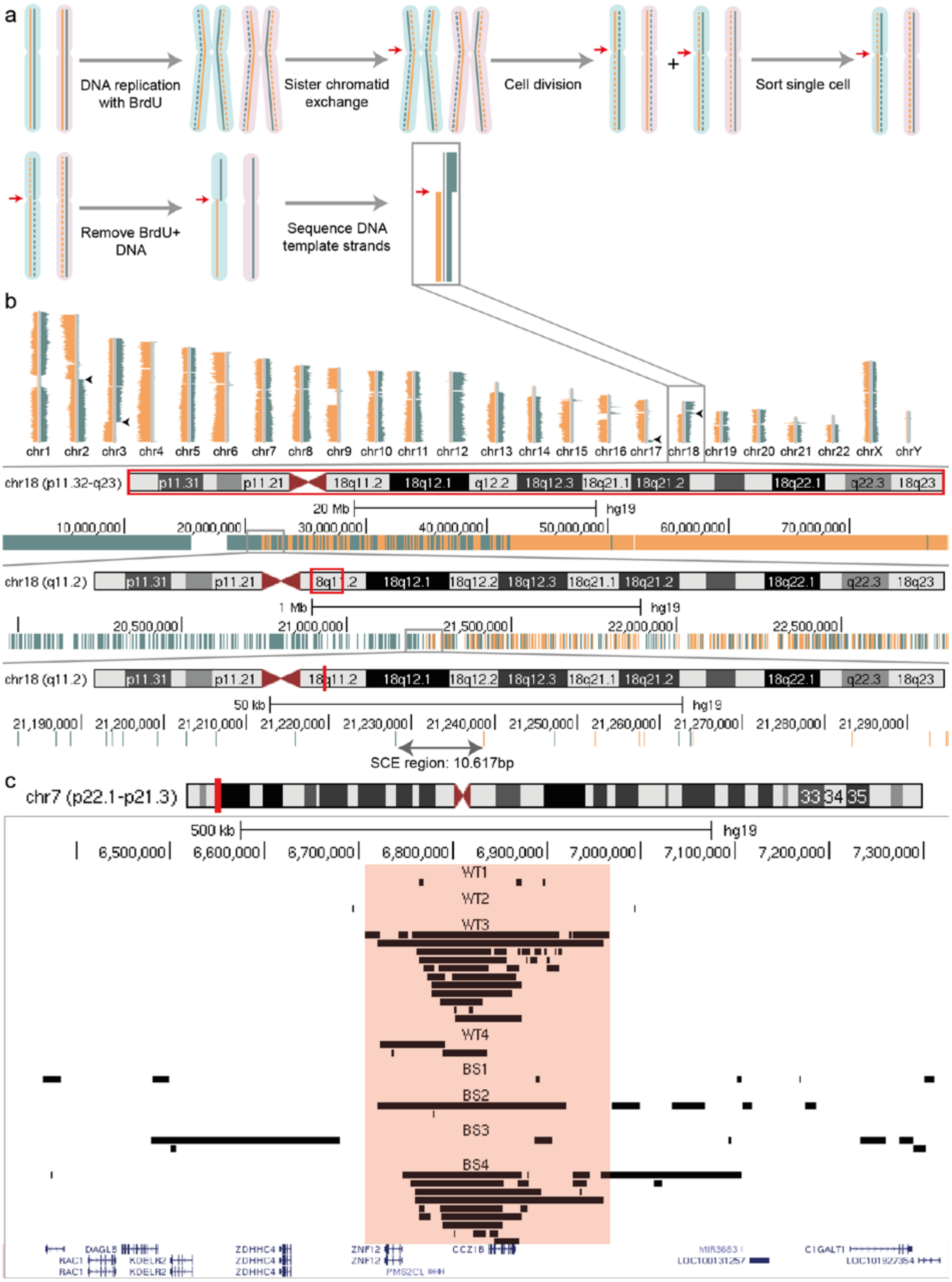
a
DNA replication with BrdU
Sister chromatid exchange
Cell division
Sort single cell
Remove BrdU+ DNA
Sequence DNA template strands
b
chr1
chr2
chr3
chr4
chr5
chr6
chr7
chr8
chr9
chr10
chr11
chr12
chr13
chr14
chr15
chr16
chr17
chr18
chr19
chr20
chr21
chr22
chrX
chrY
chr18 (p11.32-q23)
p11.31
p11.21
18q11.2
18q12.1
q12.2
18q12.3
18q21.1
18q21.2
18q22.1
q22.3
18q23
20 Mb
hg19
10,000,000
20,000,000
30,000,000
40,000,000
50,000,000
60,000,000
70,000,000
chr18 (q11.2)
1 Mb
20,500,000
21,000,000
21,500,000
22,000,000
22,500,000
50 kb
21,190,000
21,200,000
21,210,000
21,220,000
21,230,000
21,240,000
21,250,000
21,260,000
21,270,000
21,280,000
21,290,000
SCE region: 10.617bp
c
chr7 (p22.1-p21.3)
500 kb
6,500,000
6,600,000
6,700,000
6,800,000
6,900,000
7,000,000
7,100,000
7,200,000
7,300,000
WT1
WT2
WT3
WT4
BS1
BS2
BS3
BS4
RAC1
DAGLB
KDELR2
ZDHHC4
ZNF12
CCZ1B
PMS2CL
MIR3683
LOC100131257
C1GALT1
LOC101927354

Supplementary Figure 1. High resolution SCE mapping using Strand-seq, Related to Figure 1.

(a) Strand-seq allows for SCE identification as a changes in strand orientation in chromosomal DNA template strands. To this end, asynchronously cycling cells are pulsed with the thymidine-analog bromodeoxyuridine (BrdU) for one round of DNA replication and cell division. BrdU is incorporated into newly synthesized DNA strands during S-phase (dotted lines), leading to hemi-substituted sister chromatids where one sister has the template Watson strand (orange) and the other has the template Crick strand (green). An SCE event (indicated by red arrow) causes an exchange of DNA template strands, leading to both sister chromatids having a partial Watson and a partial Crick template strand. After cell division, each daughter cell has enherited one homolog where an SCE has occurred, and one unaffected homolog. At this point, single cells are sorted, BrdU-labelled DNA is selectively degraded, and the remaining DNA is sequenced. SCEs are subsequently identified and mapped as changes in DNA strand orientation from Watson to Crick, or vice versa (further explained in Supplementary Figure 1b).

(b) Example of high resolution SCE mapping in a Strand-seq library made from a BS fibroblast (same library as shown in Fig. 1b). SCE on chromosome 18 displays template strand state change from Crick-Crick (solid green) to Watson-Crick (mixed green-orange). Zooming in on approximate SCE region allows for identification of individual reads flanking the switch from Crick to Watson. The SCE is mapped to the region between these reads, in this case a 10.617bp region. The BAIT software package[56] was used for automated SCE mapping. This software calculates ratios of Watson/Crick reads in a bin (1 for 100% Watson reads, -1 for 100% Crick reads, and 0 for 50% Watson/Crick), identifies SCEs as changes in this ratio along chromosomal segments, and then localizes SCEs to the locations of the ratio switch based on the first 20 reads flanking each site of the SCE region.

(h) Example of SCE hotspot detected within FRA7B. Mapped SCE regions for each cell line were uploaded onto the UCSC Genome Browser. Black bars represent genomic locations of

SCE regions; size indicates mapping resolution using the BAIT program. Red box indicates the location of the SCE hotspot as detected by Strand-seq.

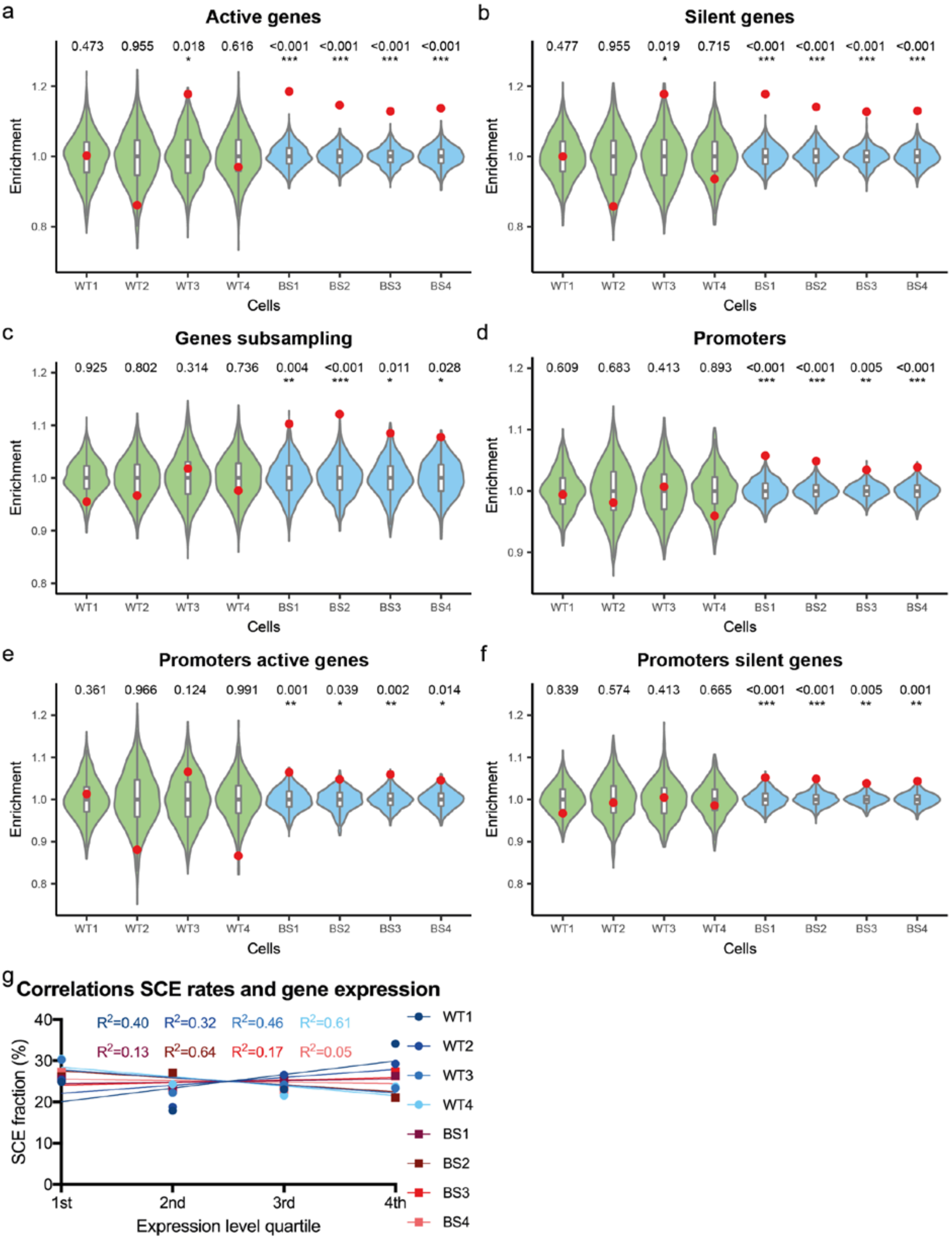
a
Active genes
0.473 0.955 0.018 0.616 <0.001 <0.001 <0.001 <0.001
* *** *** *** ***
Enrichment
1.2
1.0
0.8
WT1 WT2 WT3 WT4 BS1 BS2 BS3 BS4
Cells
b
Silent genes
0.477 0.955 0.019 0.715 <0.001 <0.001 <0.001 <0.001
* *** *** *** ***
Enrichment
1.2
1.0
0.8
WT1 WT2 WT3 WT4 BS1 BS2 BS3 BS4
Cells
c
Genes subsampling
0.925 0.802 0.314 0.736 0.004 <0.001 0.011 0.028
** *** * *
Enrichment
1.2
1.1
1.0
0.9
0.8
WT1 WT2 WT3 WT4 BS1 BS2 BS3 BS4
Cells
d
Promoters
0.609 0.683 0.413 0.893 <0.001 <0.001 0.005 <0.001
*** *** ** ***
Enrichment
1.2
1.1
1.0
0.9
WT1 WT2 WT3 WT4 BS1 BS2 BS3 BS4
Cells
e
Promoters active genes
0.361 0.966 0.124 0.991 0.001 0.039 0.002 0.014
** * ** *
Enrichment
1.2
1.1
1.0
0.9
0.8
WT1 WT2 WT3 WT4 BS1 BS2 BS3 BS4
Cells
f
Promoters silent genes
0.839 0.574 0.413 0.665 <0.001 <0.001 0.005 0.001
*** *** ** **
Enrichment
1.2
1.1
1.0
0.9
0.8
WT1 WT2 WT3 WT4 BS1 BS2 BS3 BS4
Cells
g
Correlations SCE rates and gene expression
R²=0.40 R²=0.32 R²=0.46 R²=0.61
R²=0.13 R²=0.64 R²=0.17 R²=0.05
SCE fraction (%)
40
30
20
10
0
1st 2nd 3rd 4th
Expression level quartile
WT1
WT2
WT3
WT4
BS1
BS2
BS3
BS4

Supplementary Figure 2. SCE enrichments in genes and promoters, and effect of transcriptional activity, Related to Figure 2.

Relative SCE enrichments (red points) over random distributions (violin plots) for SCEs overlapping (a) transcribed genes; (b) non-transcribed genes; (c) genes after SCE subsampling; (d) promoter regions; (e) promoter regions associated with active genes, and; (f) promoter regions associated with silent genes. SCE subsampling was achieved by randomly selecting 1559 SCEs (the number of SCEs mapped in the smallest dataset) for each randomized permutation, as well as for the overlap of experimentally determined SCE regions with genes or promoter regions. P-values indicate the fraction of permuted overlaps (out of 1.000 permutations) equal to or higher than overlap with observed SCE regions. Significant p-values are indicated as follows: *: $p<0.05$, **: $p<0.01$, ***$p<0.001$. (g) All active genes were divided into four categories of expression levels (low, medium-low, medium-high, and high) based on RPKM values. The number of SCEs overlapping all genes in each category was determined, and plotted as a fraction of the total number of SCEs overlapping active genes after correcting for the cumulative size of all genes in each category.

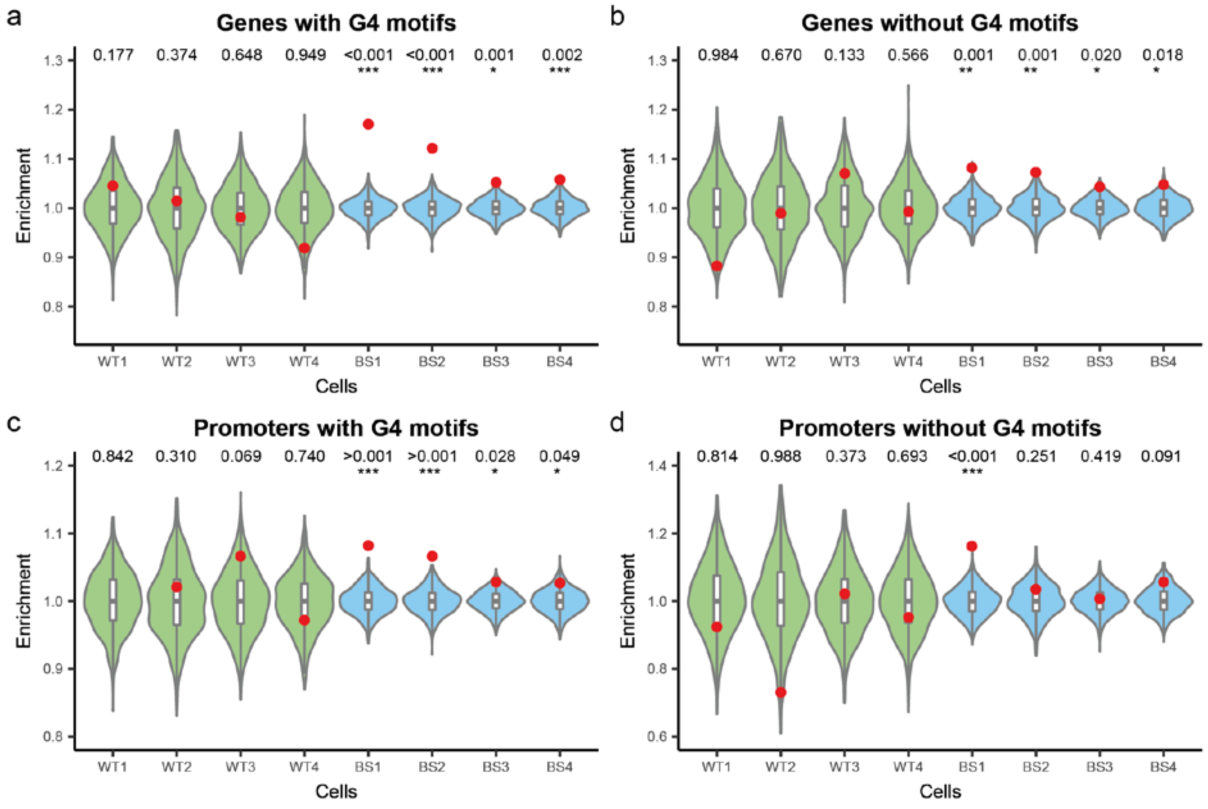

Supplementary Figure 3. SCE enrichments in genes containing and lacking G4 motifs, Related to Figure 2. Relative SCE enrichments (red points) over random distributions (violin plots) for SCEs overlapping (a) genes containing one or more G4 motifs; (b) genes lacking any G4 motifs; (c) promoters regions containing one or more G4 motifs; and (d) promoter regions lacking any G4 motifs. P-values indicate the fraction of permuted overlaps (out of 1.000 permutations) equal to or higher than overlap with observed SCE regions. Significant p-values are indicated as follows: *: $p<0.05$, **: $p<0.01$, ***$p<0.001$.

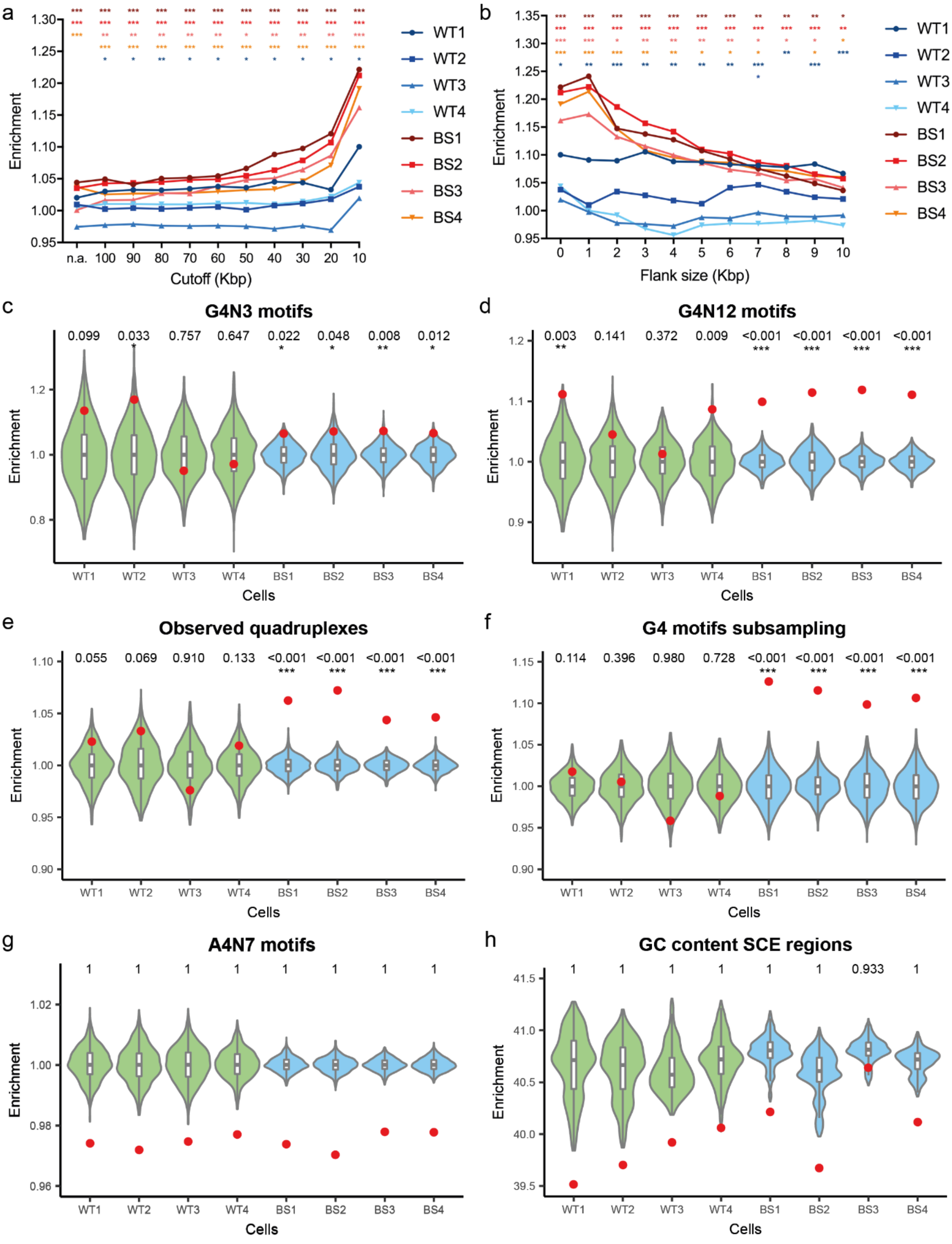

a
Enrichment
Cutoff (Kbp)
n.a. 100 90 80 70 60 50 40 30 20 10
WT1
WT2
WT3
WT4
BS1
BS2
BS3
BS4
b
Enrichment
Flank size (Kbp)
0 1 2 3 4 5 6 7 8 9 10
WT1
WT2
WT3
WT4
BS1
BS2
BS3
BS4
c
G4N3 motifs
0.099 0.033 0.757 0.647 0.022 0.048 0.008 0.012
Enrichment
Cells
d
G4N12 motifs
0.003 0.141 0.372 0.009 <0.001 <0.001 <0.001 <0.001
Enrichment
Cells
e
Observed quadruplexes
0.055 0.069 0.910 0.133 <0.001 <0.001 <0.001 <0.001
Enrichment
Cells
f
G4 motifs subsampling
0.114 0.396 0.980 0.728 <0.001 <0.001 <0.001 <0.001
Enrichment
Cells
g
A4N7 motifs
1 1 1 1 1 1 1 1
Enrichment
Cells
h
GC content SCE regions
1 1 1 1 1 1 0.933 1
Enrichment
Cells
WT1 WT2 WT3 WT4 BS1 BS2 BS3 BS4

Supplementary Figure 4. SCE enrichments at alternate G4 motifs, effect of different SCE size cutoffs, Related to Figure 2.

(a) Effect of differently size cutoffs for SCE regions included in analysis of relative SCE enrichment over median permuted overlaps. *: $p<0.05$, **: $p<0.01$, *** $p<0.001$ for color-matched cell line.

(b) Effect of adding increasingly large flanking regions to <10Kb SCE regions on relative SCE enrichment over median permuted overlaps. *: $p<0.05$, **: $p<0.01$, *** $p<0.001$ for color-matched cell line.

(c-h) Relative SCE enrichments (red points) over random distributions (violin plots) for SCEs overlapping the alternate G4 motifs (c) $G_{3+}N_{1-3}G_{3+}N_{1-3}G_{3+}N_{1-3}G_{3+}$; and (d) $G_{3+}N_{1-12}G_{3+}N_{1-12}G_{3+}N_{1-12}G_{3+}$; (e) canonical G4 motifs after subsampling SCE regions; (f) previously reported observed quadruplex forming regions; (g) $A_{3+}N_{1-7}A_{3+}N_{1-7}A_{3+}N_{1-7}A_{3+}$ motifs; (h) and GC content of all SCE regions For enrichment analyses. P-values indicate the number of permuted overlaps equal to or higher than overlap with SCE regions. SCE subsampling was achieved by randomly selecting 1559 SCEs (the number of SCEs mapped in the smallest dataset) for each randomized permutation, as well as for the overlap of experimentally determined SCE regions with genes or promoter regions. An SCE region size cutoff of 10Kb was used for these analyses. P-values indicate the fraction of permuted overlaps (out of 1.000 permutations) equal to or higher than overlap with observed SCE regions. Significant p-values are indicated as follows: *: $p<0.05$, **: $p<0.01$, ***$p<0.001$.

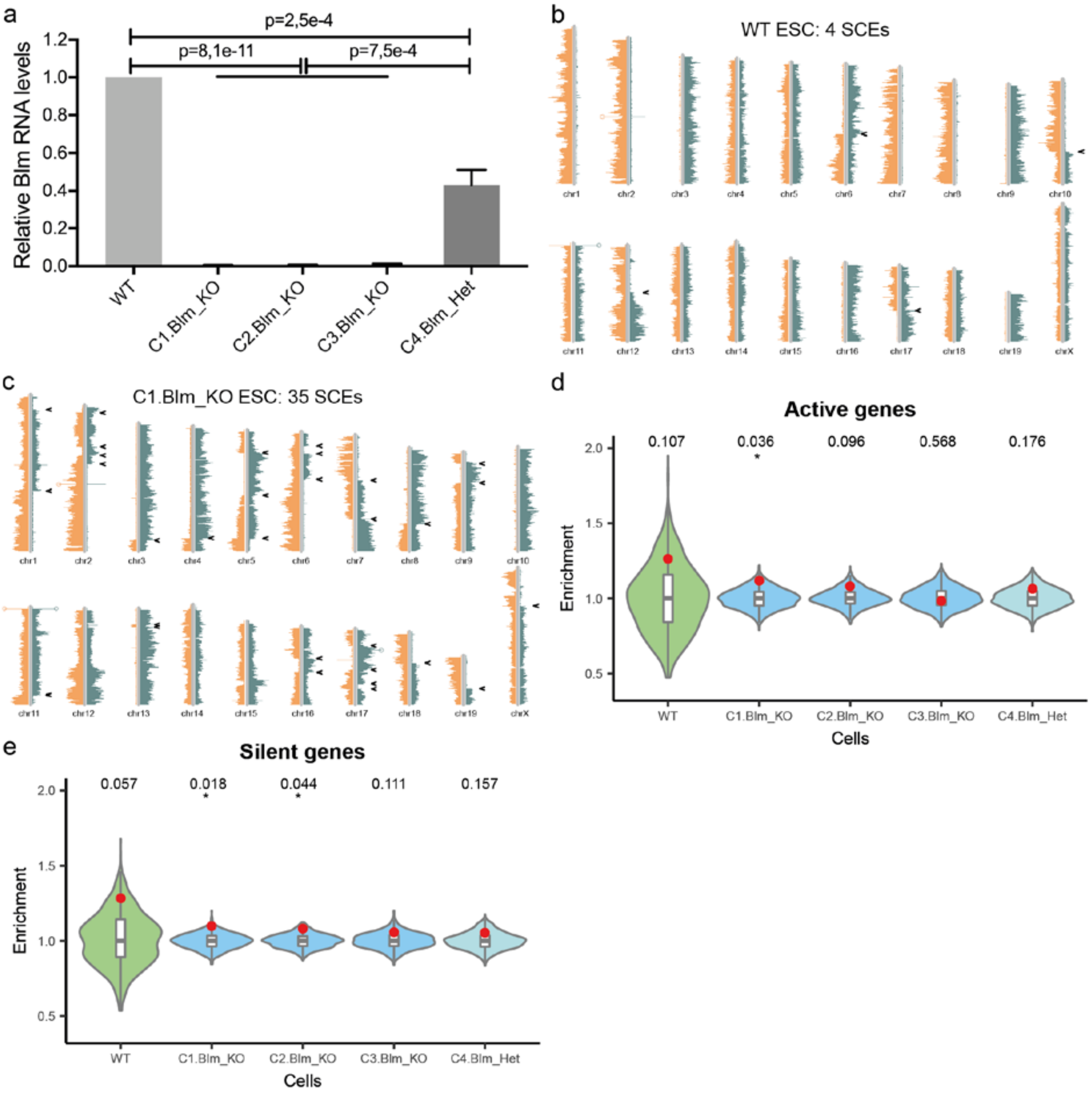

Supplementary Figure 5. Effect of *Blm* knockout on SCE rates and SCE enrichment at in genes, Related to Figure 4.

(a) qRT-PCR for Blm transcripts in WT and *Blm* mutant cell lines. Expression levels were normalized to levels in WT cells for each of three replicate experiments. P-values were calculated using t-test and ANOVA.

(b-c) Representative Strand-seq libraries generated from (b) a WT and (c) a C1.$Blm^{-/-}$ cell. Mapped DNA template strand reads are plotted on directional chromosome ideograms; Crick

(positive, sense) reads in green, Watson (negative, antisense) reads in orange. SCEs are identified as a switch in template strand state, indicated by black arrowheads.

(d-e) Relative SCE enrichments (red points) over random distributions (violin plots) for SCEs overlapping with one or multiple (d) active genes; and (e) silent genes. P-values indicate the fraction of permuted overlaps (out of 1.000 permutations) equal to or higher than overlap with observed SCE regions. Significant p-values are indicated as follows: *: $p<0.05$, **: $p<0.01$, ***$p<0.001$.

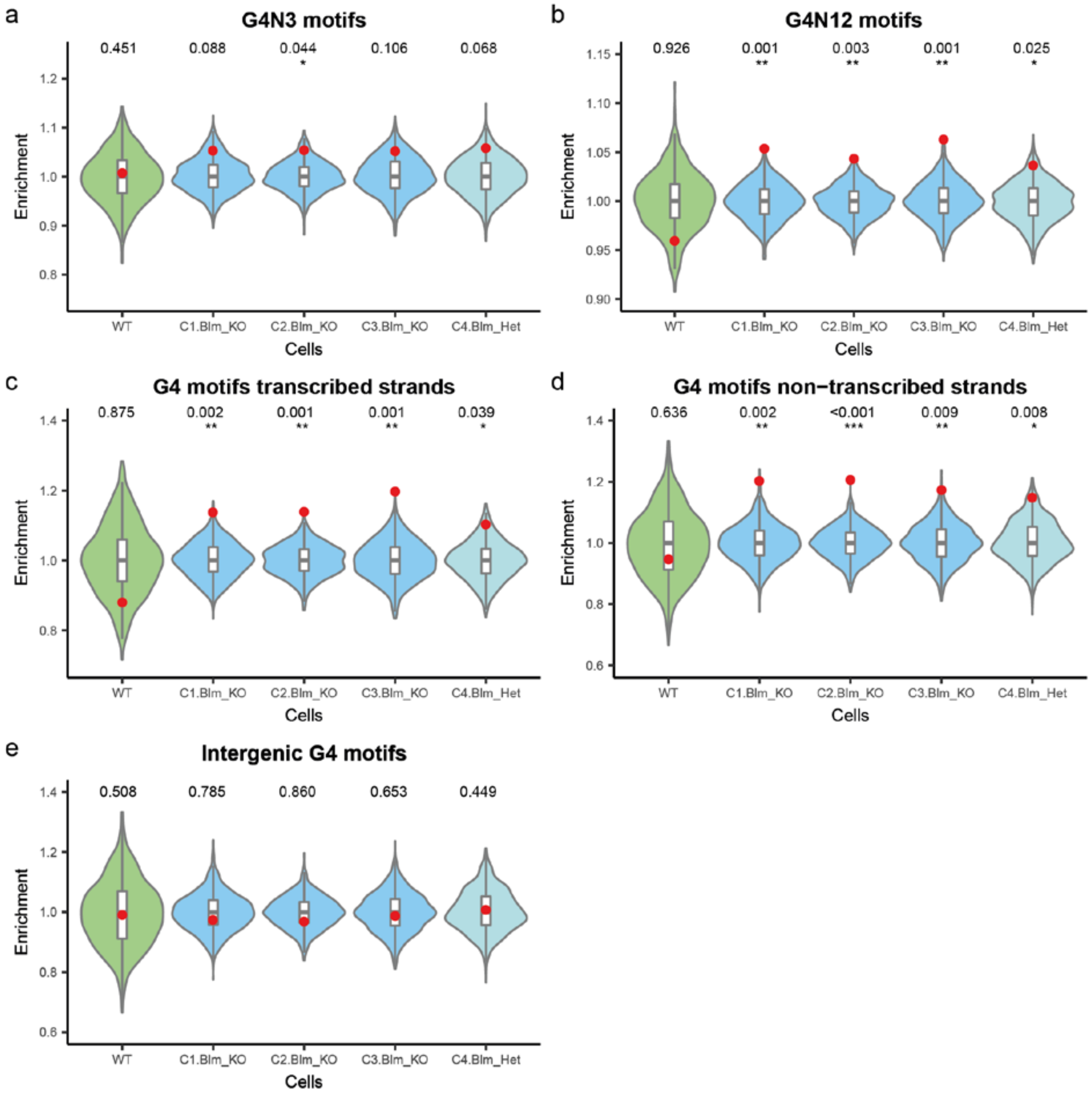

Supplementary Figure 6. Effect of *Blm* knockout on SCE enrichment at G4 motifs, Related to Figure 4.

(a-e) Relative SCE enrichments (red points) over random distributions (violin plots) for SCEs overlapping with one or multiple (a) alternate G4 motifs $G_{3+}N_{1-3}G_{3+}N_{1-3}G_{3+}N_{1-3}G_{3+}$; and (b) $G_{3+}N_{1-12}G_{3+}N_{1-12}G_{3+}N_{1-12}G_{3+}$; or canonical G4 motifs occurring in (c) intragenic transcribed strands; (d) intragenic non-transcribed strands; (e) or in intergenic regions. P-values indicate the fraction of permuted overlaps (out of 1.000 permutations) equal to or higher than overlap with observed SCE regions. Significant p-values are indicated as follows: *: $p<0.05$, **: $p<0.01$, ***$p<0.001$.

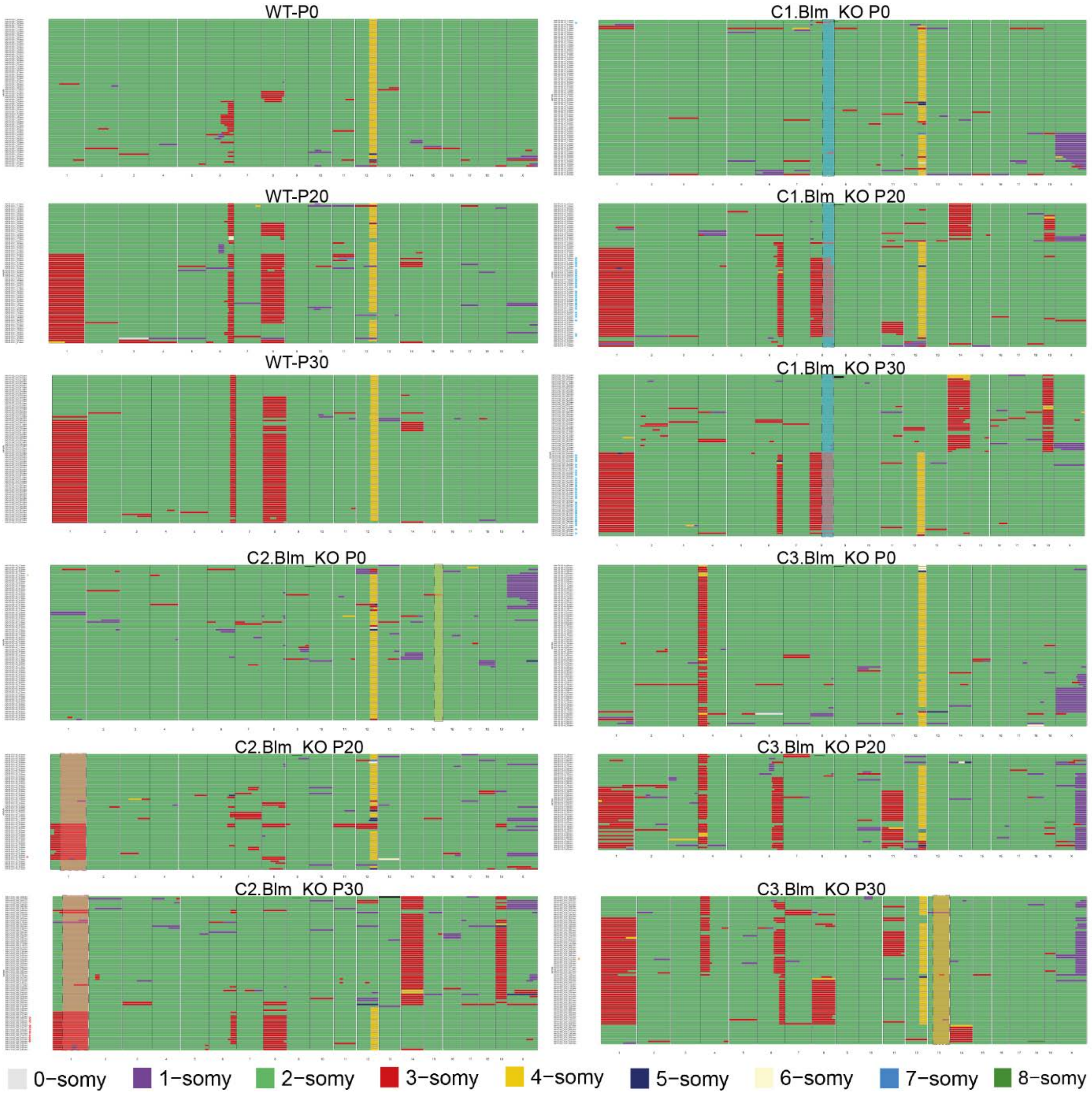

Supplementary Figure 7. Aneuploidy and CNV analysis in WT and *Blm*$^{-/-}$ ES cell lines, Related to Figure 5.

Graphical representation of chromosome copy number states in single-cell whole genome sequencing libraries prepared from WT and *Blm*$^{-/-}$ cells at indicated passage numbers. Each horizontal line represents a single-cell library, with chromosome numbers indicated below each plot. Different colors represent copy number status as detected in 2Mb bins. Cells are clustered based on the similarity of their copy number profile. LOH regions for each population are highlighted (in the same color as Figure 5a), with libraries harboring this LOH region indicated with an asterisk.

Cell line	Corriell ID	Cell type	Disease state	Strand-seq libraries (n)	SCEs (n)	Median mapping resolution (bp)	SCE regions <100 Kb (%)
WT1	GM07492	Primary fibroblast	Healthy	321	1559	15714	95.25
WT2	GM07545	Primary fibroblast	Healthy	354	1616	11430	96.93
WT3	GM12891	EBV-transformed B-lymphocyte	Healthy	334	2128	7847	96.71
WT4	GM12892	EBV-transformed B-lymphocyte	Healthy	340	2326	10722	96,28
BS1	GM02085	Primary fibroblast	Bloom Syndrome	328	11156	10102	97.58
BS2	GM03402	Primary fibroblast	Bloom Syndrome	229	8453	17497	96.05
BS3	GM16375	EBV-transformed B-lymphocyte	Bloom Syndrome	326	14667	13064	96.94
BS4	GM17361	EBV-transformed B-lymphocyte	Bloom Syndrome	306	12724	10805	95.99

Supplementary Table 1. Overview of human cell lines used, Strand-seq libraries made, and SCEs identified and mapped, Related to Figures 1-3.

Clone	sgRNAs	Allele	Deletion coordinates	Strand-seq libraries (n)	SCEs (n)	Median mapping resolution (bp)	SCE regions <100 Kb (%)
WT	n.a.	Cast	Not mutated	98	244	8048	99.16
		129	Not mutated				
C1.Blm_KO	1+2	Cast	71163-71636	91	3048	7392	98.59
		129	71167-71396				
C2.Blm_KO	3+4	Cast	71166-72195	97	3801	6251	98.01
		129	71166-72195				
C3.Blm_KO	1+2	Cast	71161-71570	67	2597	6722	98.51
		129	71161-71570				
C4.Blm_Het	3+4	Cast	71377-71848	107	2437	7554	97.58
		129	Not mutated				

Supplementary Table 2. Overview of *Blm* mutant mouse ES cell lines generated and used for this study, Related to Figures 4-5.
